# Cricothyroidotomy for Can’t Intubate, Can’t Oxygenate Situation in Emergency Department

**DOI:** 10.31729/jnma.v63i2091.9231

**Published:** 2025-11-30

**Authors:** Kripa Maharjan, Dipesh Mangal Joshi, Ashis Shrestha

**Affiliations:** 1 Patan Academy of Health Sciences, Lagankhel, Lalitpur, Nepal

**Keywords:** *airway management*, *cricothyroidotomy*, *CICO*, *emergency medicine*, *scalpel bougie technique*

## Abstract

Airway emergencies represent some of the most time-critical and high-stress situations in emergency and critical care practice. Cricothyroidotomy, though infrequently performed, remains the definitive lifesaving procedure when conventional airway management fails in a “cannot intubate, cannot oxygenate” (CICO) scenario. This viewpoint discusses a real-life experience of performing bougie-assisted cricothyroidotomy in a patient with tetanus-induced trismus, reviews the technical aspects of the procedure, and highlights the importance of preparedness, simulation training, and equipment readiness. Despite its rarity, the procedure’s successful execution can be the difference between life and death, emphasizing the need for continuous skill maintenance among emergency and critical care professionals.

## INTRODUCTION

Airway emergencies are among the most challenging and time-sensitive events faced in the emergency department. The “can’t intubate, can’t oxygenate” (CICO) situation represents one of the most critical crises in airway management, where seconds determine survival.^1^ Despite extensive training in airway-algorithms, the transition from simulation to real-life execution can be profoundly challenging. Emergency departments (EDs), where patient presentations are unpredictable and often rapid-lyprogressive, remain a high-risk setting for such events.^2^ This viewpoint shares a firsthand experience of managing an unanticipated airway emergency that reinforced the principle—always prepare for the worst.

## EXPERIENCE

Our team encountered a CICO situation in a patient with suspected tetanus who developed trismus and generalized muscle spasm and then had cardiac arrest. Conventional airway maneuvers and ventilation were impossible, requiring Cricothyroidotomy. Despite successfully establishing an airway and initiating resuscitation, the patient did not survive. This case highlights the unpredictable nature of emergency medicine, where a seemingly stable patient can deteriorate catastrophically in seconds. The rapid onset of rigidity and ventilatory failure in this patient suggests possible etiologies such as malignant hyperthermia, tetanic seizure, neuroleptic malignant syndrome, hypocalcemia, or toxin-induced rigidity. Without postmortem analysis, the cause remains speculative.

## CRICOTHYROIDOTOMY

Though rare, CICO events are universally feared. Their reported incidence in anesthesia settings is approximately 1 in 10,000 to 1 in 50,000 cases, but may be higher in the ED due to lack of preoptimization and emergent circumstances.^2^ Successful management requires rapid identification of failure and immediate escalation to a surgical airway without hesitation. Delay in decision-making is a frequent contributor to mortality. Cricothyroidotomy is often described as a “once-in-a-lifetime” procedure for emergency physicians.^3^ While training on mannequins and cadavers builds familiarity, the emotional and practical challenges of performing it on a real patient are incomparable. The tactile sensation, time pressure, and psychological intensity cannot be replicated in simulation.^4^ In this case, despite the patient’s death, successful establishment of a surgical airway was a critical step, reaffirming the importance of readiness.

## SCALPEL BOUGIE TECHNIQUE

The bougie-assisted cricothyroidotomy is a simple, rapid, and reliable method for securing an airway in emergencies. The patient is placed supine with mild neck extension, and the cricothyroid membrane is palpated between the thyroid and cricoid cartilages. After preparing sterile equipment, a number 11 scalpel blade, gum elastic bougie, cuffed 6.0 mm endotracheal tube, lubricant, and antiseptic, a 2-3 cm vertical skin incision is made over the membrane to improve exposure. A horizontal stab incision is then made through the membrane, creating entry into the airway.^5^

The bougie is gently advanced into the trachea, and correct placement can be confirmed by feeling tracheal rings or a “hold-up” sensation. A lubricated cuffed endotracheal tube is railroaded over the bougie into the trachea and advanced until the cuff passes through the membrane. After inflating the cuff, ventilation is initiated and placement confirmed by capnography and auscultation.

A 6.0 mm cuffed endotracheal tube is generally preferred for balancing ease of insertion and adequate ventilation. Oversized tubes risk damaging nearby structures, including the cricoid cartilage and tracheal rings.^6^

This method is favored for its speed, simplicity, and lower risk of false passage. When performed efficiently, airway access can be established within 60 seconds, minimizing hypoxic injury. Nevertheless, potential complications include misplacement, bleeding, infection, or injury to adjacent structures, especially if performed under stress or delay.

## EMOTIONAL IMPACT AND REFLECTION

The inability to save a patient, especially when the cause remains elusive, leaves a lasting impression. The event reinforces humility and emphasizes the essence of emergency medicine—decisive action under uncertainty. Performing a cricothyroidotomy, even once, profoundly changes a physician’s perspective on airway preparedness.^7^ It is a reminder that in medicine, success lies not only in outcomes but in readiness to act when there is no alternative. Preparedness through simulation, cadaveric dissection, and interprofessional drills are vital to maintaining procedural readiness. It is equally important that institutions should adopt structured simulation programs and maintain standardized CICO kits to improve outcomes in airway crises.

## CONCLUSION

This experience emphasizes the core principle of emergency medicine: always prepare for the worst. Airway catastrophes can occur unexpectedly, and every second counts. Mastery of the cricothyroidotomy procedure—through training, repetition, and mental rehearsal—remains vital. Mastery of this technique may rarely be called upon but when it is, hesitation can cost lives.
